# Gentisic acid prevents the transition from pressure overload-induced cardiac hypertrophy to heart failure

**DOI:** 10.1038/s41598-019-39423-8

**Published:** 2019-02-28

**Authors:** Simei Sun, Hae Jin Kee, Yuhee Ryu, Sin Young Choi, Gwi Ran Kim, Hyung-Seok Kim, Seung-Jung Kee, Myung Ho Jeong

**Affiliations:** 1 Zhoushan Hospital, Zhejiang University School of Medicine, No 739, Dingshen Road Lincheng New District, Zhoushan Zhejiang, 316021 China; 2 Heart Research Center of Chonnam National University Hospital, Gwangju, 61469 Republic of Korea; 3 Hypertension Heart Failure Research Center, Chonnam National University Hospital, Gwangju, 61469 Republic of Korea; 4 Molecular Medicine, BK21 plus, Chonnam National University Graduate School, Gwangju, 61469 Republic of Korea; 5 Department of Forensic Medicine, Chonnam National University Medical School, Gwangju, 61469 Republic of Korea; 6 Department of Laboratory Medicine, Chonnam National University, Medical School and Hospital, Gwangju, 61469 Republic of Korea

## Abstract

We previously reported that gentisic acid attenuates cardiac hypertrophy and fibrosis in transverse aortic constriction (TAC)-induced cardiac hypertrophy. Here, we examined whether gentisic acid prevents the development of heart failure. Heart failure was induced in mice via chronic TAC. Mice were administered the vehicle, gentisic acid (10 and 100 mg∙kg^−1^∙day^−1^), or bisoprolol (0.5 mg∙kg^−1^∙day^−1^) orally for 3 weeks, beginning 3 weeks after TAC. After oral administration of gentisic acid (2000 mg∙kg^−1^), no significant differences in organ weight, histology, or analyzed serum and hematological parameters were observed between female mice in the control and gentisic acid-treated groups. Gentisic acid administration inhibited cardiac dysfunction in a dose-dependent manner, and reduced cardiac hypertrophy and fibrosis, as was revealed via western blotting, quantitative real-time PCR, and Masson’s trichrome staining. Gentisic acid dose-dependently reduced the expression of fibrosis marker genes, suppressed the renin-angiotensin-aldosterone system, and reduced lung size and pulmonary vascular remodeling. Our data indicate that gentisic acid prevents cardiac hypertrophy, fibrosis, cardiac dysfunction, and pulmonary pathology in TAC-induced heart failure. These findings suggest that supplementation with gentisic acid may provide an advantage in preventing the progression from cardiac hypertrophy to heart failure.

## Introduction

Heart failure is a functioning abnormality caused by reduced contraction of the heart, or an abnormality of the heart structure^1^. It is induced by a variety of diseases, such as myocardial infarction, hypertension, aortic stenosis, and valvular heart disease, which are more prevalent in people aged 65 or older^2–4^. However, recently, the relationship between heart failure and the activation of the sympathetic nervous system (SNS) and the renin-angiotensin-aldosterone system (RAAS) has attracted attention^5–7^. Heart failure is usually accompanied by cardiac hypertrophy and fibrosis^8^. Initially, the heart has a reward mechanism such as a cardiac hypertrophy, but continuous stress causes myocardial apoptosis and interstitial fibrosis, leading to ventricular dilatation^9^. Dyspnea is a common symptom in patients with heart failure owing to congestive symptoms of the lungs^10^. Angiotensin-converting enzyme (ACE), angiotensin receptor blockers (ARB), beta-blockers (BB), and mineralocorticoid receptor antagonists are used in the treatment of heart failure^11–13^. However, the mortality rate is relatively high as this treatment is imperfect, and it is therefore necessary to develop new heart failure treatments as well as preventive drugs or natural substances.

Hydroxybenzoic acids are phytochemicals that are related to salicylic acid. Gentisic acid, or 2,5-dihydroxybenzoic acid, is one such acid, and is produced by plants such as kiwi^14^, melon^15^, and *Dendropanax morbifera*^16^, to protect themselves from external infections^17^.

Gentisic acid exerts several beneficial effects on heart attacks, atherogenesis^18^, and lipid hydroperoxide production^19^. It is also known as a fibroblast growth factor inhibitor that can inhibit tumor growth^20^.

We previously demonstrated that gentisic acid attenuates pressure overload-induced cardiac hypertrophy and fibrosis in mice^21^. It is not yet known whether gentisic acid affects heart failure; we hypothesized that it could prevent heart failure. To explore this idea, we compared the effects of treatment with the BB bisoprolol and treatment with two concentrations of gentisic acid in a mouse model of transverse aortic constriction (TAC)-induced heart failure.

## Materials and Methods

### TAC and experimental groups

All animal procedures were approved by the Animal Experimental Committee of Chonnam National University Medical School (CNU IACUC-H-2018-4), and were carried out according to the Guide for the Care and Use of Laboratory Animals (US National Institutes of Health Publications, 8^th^ edition, 2011). CD-1 male mice (6 weeks old, weighing approximately 30 g) were anesthetized via intraperitoneal injection of ketamine (120 mg∙kg^−1^) and xylazine (6.2 mg∙kg^−1^). Mice underwent either sham surgery or TAC. The mice’s endotracheal tubes were connected to a rodent ventilator, and the thymus was removed after exposure of the aortic arch. The transverse aortic arch was ligated (using a 7-0 silk suture) between the brachiocephalic and left common carotid arteries with an overlaying 27 G needle. Mice in the control group underwent the same operation, but without ligation of the aorta. Success of the TAC procedure was confirmed via echocardiography. After 3 weeks, drugs were orally administered to mice for a further 3 weeks. The mice were then divided into five groups: sham (n = 10), TAC (n = 9), TAC + gentisic acid (n = 10, 10 mg∙kg^−1^∙day^−1^), TAC + gentisic acid (n = 10, 100 mg∙kg^−1^∙day^−1^), and TAC + bisoprolol (n = 10, 0.5 mg∙kg^−1^∙day^−1^). Gentisic acid and bisoprolol were dissolved in distilled water (DW). Tibia length (TL) was determined and the ratio of heart weight (HW) to body weight (BW) (i.e., HW/BW) was calculated. In addition, the ratios of lung weight (LW) to BW (i.e., LW/BW) and LW to TL (i.e., LW/TL) were calculated.

### Echocardiography

Echocardiography was performed using a Vivid S5 echocardiography system (GE Healthcare, Chicago, IL, USA) with a 13-MHz linear array transducer. Mice were anesthetized using tribromoethanol (Avertin, 114 mg∙kg^−1^ intraperitoneal injection) before the procedure. M-mode (2-D guided) images and recordings were acquired from the long-axis view of the left ventricle at the level of the papillary muscles. The thickness of the anterior and posterior walls was measured from the images, and the left ventricular end-diastolic diameter (LVEDD) and left ventricular end-systolic diameter (LVESD) were measured from the M-mode recordings. Fractional shortening (FS) was calculated as FS (%) = (LVEDD - LVESD) × 100/LVEDD. The ejection fraction (EF) was calculated as EF (%) = (stroke volume (SV)/end-diastolic volume (EDV)) × 100.

### Acute toxicity test and organ weight

The acute toxicity study was conducted in accordance with the Organization for Economic Co-operation and Development (OECD) 420 Guideline for Testing of Chemicals. Twelve female mice were randomly assigned to two groups (n = 6): the control group (which received tap water) and the treatment group (which received fixed doses of gentisic acid, i.e., 2000 mg∙kg^−1^ BW, diluted in DW). All administrations were performed orally on the first day of the experiment only. The mice were monitored for 14 days and changes in the general physical conditions including appearance, fur condition, behavior, and mortality were recorded. BW was measured once a week using a tabletop electronic balance. After sacrifice, the liver, lungs, kidneys, and heart were weighed. The ratio of organ weight to BW was evaluated.

### Serum biochemical analysis and hematological assessment

Blood was collected in tubes containing anticoagulants, and the biochemical analysis of blood was commissioned to the Korea Testing & Research Institute (KTR, Hwasun, South Korea). Blood samples (0.8–0.9 mL) were collected via cardiac puncture using a disposable syringe in K2EDTA tubes for hematological parameter analysis, and in plain tubes for biochemical parameter assessment. The biochemical parameters assessed were total protein (TP), albumin (ALB), globulin, albumin/globulin ratio (A/G), total bilirubin (T-BIL), alkaline phosphatase (ALP), aspartate aminotransferase (AST), alanine aminotransferase (ALT), creatinine, and blood urea nitrogen (BUN). Furthermore, the numbers of white blood cells (WBC), neutrophils, lymphocytes, monocytes, eosinophils, basophils, red blood cells (RBC), and platelets (PLT) were counted, and hemoglobin (HGB), hematocrit (HCT), mean corpuscular volume (MCV), mean corpuscular hemoglobin (MCH), mean corpuscular hemoglobin concentration (MCHC) were determined.

### Hematoxylin and eosin (H&E) staining

To investigate lung histological changes resulting from heart failure and acute toxicity, paraffin-embedded tissues were cut transversely into 3-µm-thick sections, deparaffinized with xylene, and then rehydrated with different grades of alcohol before staining with H&E, as previously described^22^. Photomicrographs were obtained using an Eclipse Ti-U microscope (Nikon, Tokyo, Japan).

### Masson’s trichrome staining

Masson’s trichrome staining was performed according to a method previously described^23^ to evaluate interstitial and perivascular collagen deposition in the heart and lungs. Collagen was stained blue. To quantify the degree of fibrosis, the NIS Elements Software was used (Nikon Eclipse 80*i*; Nikon).

### Reagents

Gentisic acid (149357) was purchased from Sigma-Aldrich (St. Louis, MO, USA). Bisoprolol fumarate (S1206) was purchased from Selleck Chemicals (Houston, TX, USA).

### Real-time reverse transcription-polymerase chain reaction (RT-PCR)

Total RNA was isolated from heart tissue using TRIzol reagent (Invitrogen Life Technologies, Carlsbad, CA, USA), and 1 μg RNA was used for the reverse transcription reaction with TOPscript RT DryMIX (Enzynomics, Daejeon, South Korea). mRNA levels were quantified using a SYBR Green PCR kit (Enzynomics). The PCR primers used in this study are listed in Table 1.

**Table 1:** Primers for RT-PCR.

Gene	Primer sequence (5′ to 3′)
*GAPDH (mouse)*	F: GCATGGCCTTCCGTGTTCCT
	R: CCCTGTTGCTGTAGCCGTATTCAT
*Collagen I (mouse)*	F: GAGCGGAGAGTACTGGATCG
	R: GCTTCTTTTCCTTGGGGTTC
*SMA (mouse)*	F: ACTGGGACGACATGGAAAAG
	R: AGAGGCATAGAGGGACAGCA
*Fibronectin (mouse)*	F: GATGCACCGATTGTCAACAG
	R: TGATCAGCATGGACCACTTC
*CTGF (mouse)*	F: CAAAGCAGCTGCAAATACCA
	R: GGCCAAATGTGTCTTCCAGT
*Collagen III (mouse)*	F: TGATGGAAAACCAGGACCTC
	R: CAGTCTCCCCATTCTTTCCA
*TGF-β1 (mouse)*	F: TTGCTTCAGCTCCACAGAGA
	R: TGGTTGTAGAGGGCAAGGAC
*ANP (mouse)*	F: TGGAGGAGAAGATGCCGGTAGAAGAT
	R: AGCGAGCAGAGCCCTCAGTTTGCT
*BNP (mouse)*	F: CTGAAGGTGCTGTCCCAGAT
	R: GTTCTTTTGTGAGGCCTTGG
*Skeletal α-actin (mouse)*	F: CGACATCAGGAAGGACCTGT
	R: ACATCTGCTGGAAGGTGGAC
*LOX (mouse)*	F: AATCCAATGGGAGAACAACG
	R: GTTGTCACGCAGCAGAAGAA
*LOXL1 (mouse)*	F: CCGACAACTGGAGAGAGGTG
	R: CGTAGCCCTGTTCGTAGGTC
*LOXL2 (mouse)*	F: GCTACGTGGAGGTGAAGGAG
	R: CTTCCAGTAACGCAGCTTCC
*CCN5 (mouse)*	F: ATACAGGTGCCAGGAAGGTG
	R: GTTGGATACTCGGGTGGCTA
*Smad7 (mouse)*	F: CAGCTCAATTCGGACAACAA
	R: GATGGAGAAACCAGGGAACA
*Syndecan 4 (mouse)*	F: ATCTGGATGACACGGAGGAG
	R: GGCCCTTTTAGGAATGACCT
*Periostin (mouse)*	F: AATGCTGCCCTGGCTATATG
	R: TTCTCCAGTCCTCTGCGAAT

### Western blotting

Total protein was extracted from heart tissues using a lysis buffer (radioimmunoprecipitation assay (RIPA) buffer, 150 mM NaCl, 1% Triton X-100, 1% sodium deoxycholate, 50 mM Tris-HCl pH 7.5, 2 mM EDTA, 1 mM PMSF, 1 mM DTT, 1 mM Na_3_VO_4_, and 5 mM NaF) containing a protease inhibitor cocktail (Calbiochem, EMD Millipore, Billerica, MA, USA). Proteins were subjected to SDS-PAGE and transferred to polyvinylidene difluoride (PVDF) membranes. The membranes were blocked with 5% skim milk in Tris-buffered saline with Tween® 20 (TBST) buffer (20 mM Tris, 200 mM NaCl, and 0.04% Tween® 20) for 1 h at 25 °C. The membranes were incubated overnight at 4 °C with the indicated primary antibodies. Antibodies for connective tissue growth factor (CTGF, sc-101586), fibronectin (sc-59826), smooth muscle alpha actin (SMA, sc-130617), transforming growth factor beta-1 (TGF-β1, sc-146), ACE (sc-20791), angiotensin II receptor type 1 (AT1, sc-515884), endothelin-1 receptor type A (ETAR, sc-135902), and glyceraldehyde 3-phosphate dehydrogenase (GAPDH, sc-32233) were purchased from Santa Cruz Biotechnology (Dallas, TX, USA). Antibodies for atrial natriuretic peptide (ANP, GTX109255) were purchased from Genetex (Irvine, CA, USA). These were incubated with anti-rabbit or anti-mouse horseradish-peroxidase-conjugated secondary antibodies (1:5000) for 1 h at 25 °C. Protein bands were visualized using Immobilon Western detection reagents (EMD Millipore). The Bio-ID software was used to quantify protein expression (Vilber Lourmat, Eberhardzell, Germany).

### Statistical analysis

All data are expressed as means ± standard error of the mean (SEM). Differences between data were analyzed via one-way analysis of variance (ANOVA) with Bonferroni post-hoc test using GraphPad Prism version 5 (GraphPad Software Inc., San Diego, CA, USA), and a value of *P* < 0.05 was considered significant.

## Results

### Effects of gentisic acid on tissue weight, biochemistry, and hematology in an acute toxicity test

**Figure 1:**
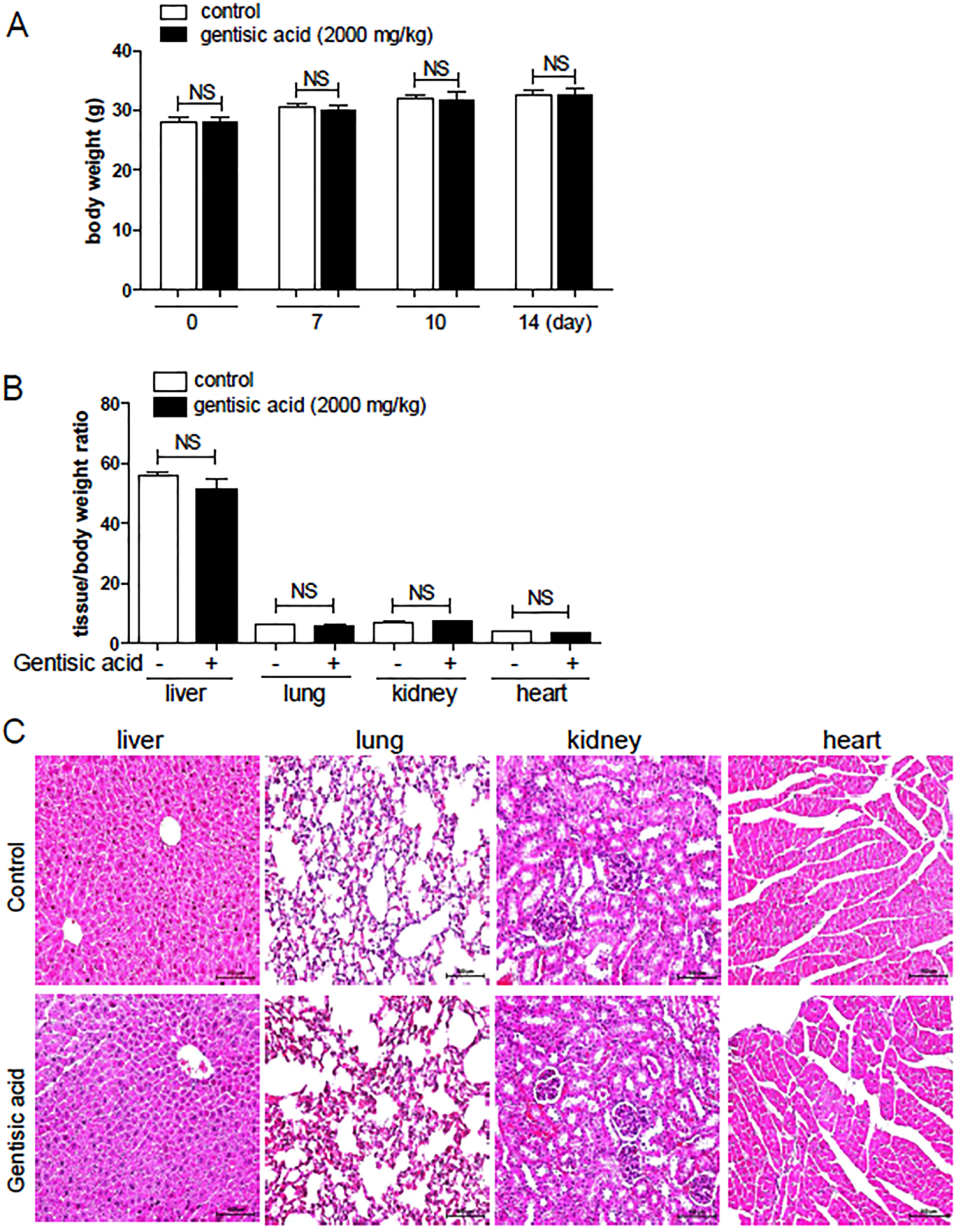
Body weight, relative organ weight ratio, and histology in acute oral toxicity study. Female CD-1 mice received water or a single dose of gentisic acid (2000 mg∙kg^−1^) orally. (**A**) Body weight was measured just before drug administration and on 7, 10, and 14 days after administration. NS: not significant. (**B**) After 14 days, the weights of the liver, lungs, kidneys, and heart were measured and expressed as the ratio of organ weight to body weight. NS: not significant. (**C**) Representative histological images of four tissues stained with hematoxylin and eosin (H&E). Scale bar = 100 μm.

Acute toxicity tests were conducted to provide clinical evidence for the use of gentisic acid in the treatment of heart failure. Gentisic acid was administered to female mice once at an oral dose of 2000 mg/kg, and mortality, BW, and behavioral changes were recorded over 14 days. One mouse died on the first day of administration in the gentisic acid treatment group. The difference in body weight between the two groups was not different throughout the experiment (0, 7, 10, 14 days). The control and the gentisic acid treatment groups showed a slight increase in body weight over time (Fig. 1A).

After sacrificing the mice on the 15^th^ day, the weight of the tissues was measured. The weight of the liver, lungs, kidneys, and heart did not differ between the two groups (Fig. 1B). H&E staining revealed no abnormal morphological changes in any of the tissues (Fig. 1C). We then performed a biochemical analysis of the blood. There were no differences in the levels of TP, albumin, globulin, T-BIL, AST, and creatinine between the two groups (Table 2). The A/G ratio, and levels of ALP and BUN in the sham and gentisic acid treatment groups showed significant differences (Table 2). In addition, we examined hematological characteristics. The WBC and RBC counts did not differ between the two groups; however, the number of basophils was significantly increased in the gentisic acid treatment group compared with that in the control group (Table 3). HGB and HCT levels showed a significant increase in the gentisic acid-treated group relative to those in the control group (Table 3). The MCHC was lower in the gentisic acid-treated group than in the sham group. The platelet number was significantly decreased in the gentisic acid treatment group.

**Table 2:** Serum biochemical parameters of female mice treated with oral gentisic acid.

Indices	TP (g/dL)	Albumin (g/dL)	Globulin (g/dL)	A/G	T-BIL (mg/dL)	ALP (U/L)	AST (U/L)	ALT (U/L)	Cr (mg/dL)	BUN (mg/dL)	BUN/Cr ratio
Control (DW) n = 6	5.4 ± 0.26	3.3 ± 0.19	2.1 ± 0.26	1.8 ± 0.05	0.04 ± 0.02	177.5 ± 10.78	144.7 ± 27.10	43.5 ± 6.28	0.3 ± 0.05	20.0 ± 1.19	76.5 ± 16.41
Gentisic acid (2000 mg/kg) n = 5	5.6 ± 0.11	3.3 ± 0.14	2.2 ± 0.07	1.6 ± 0.12*	0.03 ± 0.01	271.4 ± 12.46***	172.4 ± 30.47	44.0 ± 10.79	0.3 ± 0.04	18.0 ± 0.48**	57.4 ± 8.79

Values are means ± SEM (n = 6 mice per group).

Abbreviations: DW, distilled water; TP, total protein; A/G, albumin/globulin ratio; T-BIL, total bilirubin; ALP, alkaline phosphatase; AST, aspartate aminotransferase; ALT, alanine aminotransferase; Cr, creatinine; BUN, blood urea nitrogen.

*P < 0.05, **P < 0.01, and ***P < 0.001 versus the control.

**Table 3:** Hematological analysis of female mice treated with oral gentisic acid.

Indices	WBC (10^3^ cells/μL)	WBC difference (%)					RBC (10^6^ cell/μL)	HGB (g/dL)	HCT (%)	MCV (fL)	MCH (pg)	MCHC (g/dL)	PLT (10^3^ cells/μL)
		Neut	Lymph	Mono	Eos	Baso							
Control (DW) n = 6	1.13 ± 0.10	21.02 ± 2.23	71.62 ± 1.26	1.05 ± 0.38	3.37 ± 1.02	1.65 ± 0.66	8.59 ± 0.38	13.65 ± 0.22	47.58 ± 1.01	58.03 ± 1.12	16.60 ± 0.36	28.55 ± 0.54	893.75 ± 50.89
Gentisic acid (2000 mg/kg) n = 5	1.30 ± 0.39	18.16 ± 3.25	71.90 ± 1.95	1.36 ± 0.21	3.12 ± 0.79	2.73 ± 0.33*	8.73 ± 0.29	14.2 ± 0.44*	50.64 ± 2.58*	58.98 ± 1.71	16.28 ± 0.59	27.26 ± 0.30**	751.5 ± 76.32*

Values are means ± SEM (n = 6 mice per group).

Abbreviations: DW, distilled water; WBC, white blood cells; Neut, neutrophils; Lymph, lymphocytes; Mono, monocytes; Eos, eosinophils; Baso, basophils; RBC, red blood cells; HGB, hemoglobin; HCT, hematocrit; MCV, mean corpuscular volume; MCH, mean corpuscular hemoglobin; MCHC, mean corpuscular hemoglobin concentration; PLT, platelets.

*P < 0.05 and **P < 0.01 versus the control.

### Gentisic acid recovered cardiac dysfunction and suppressed cardiac hypertrophy in TAC-induced heart failure

**Figure 2:**
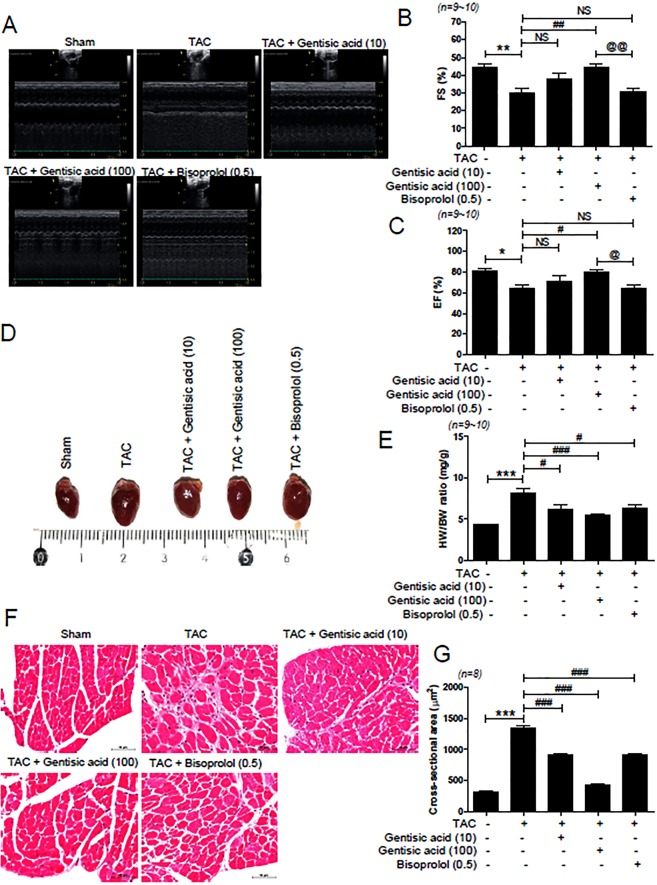
Gentisic acid prevented cardiac dysfunction and suppressed cardiac hypertrophy in transverse aortic constriction (TAC)-induced heart failure. (**A**) Representative M-mode echocardiography images in sham-operated mice, mice subjected to TAC, and mice subjected to TAC and treated with gentisic acid (10 mg∙kg−1∙day−1 or 100 mg∙kg−1∙day−1) or bisoprolol (0.5 mg∙kg-1∙day-1). (**B**) Fractional shortening (FS) and (**C**) ejection fraction (EF). Data shown are means ± SEM, n = 9–10 for each group. *P < 0.05 and **P < 0.01 versus the sham group; ^#^P < 0.05 and ^##^P < 0.01 versus the TAC group; ^@^P < 0.05 and ^@@^P < 0.01 versus bisoprolol-treated TAC group; NS: not significant. (**D**) Whole-heart photograph of mice in the sham, TAC, TAC + gentisic acid (10 mg∙kg−1∙day−1), TAC + gentisic acid (100 mg∙kg−1∙day−1), or TAC + bisoprolol (0.5 mg∙kg−1∙day−1) groups. (**E**) Heart weight to body weight ratio (HW/BW) was evaluated 6 weeks after TAC (n = 9–10 per group). (**F**) Representative images of H&E-stained, gentisic acid or bisoprolol-treated TAC hearts. Scale bar = 50 μm. (**G**) Cross-sectional areas of cardiomyocytes were quantified (n = 8 per group). ***P < 0.001 versus the sham group; ^###^P < 0.001 versus the TAC group.

To investigate whether gentisic acid treatment affects the function of the heart in a TAC mouse model, echocardiography was performed. Six weeks after TAC, FS and EF values were significantly reduced and there were no differences between the sham group and the 100 mg gentisic acid-treated group (Fig. 2A–C). However, the TAC-associated reduced FS and EF values in the 0.5 mg bisoprolol treatment group and the 10 mg gentisic acid-treatment group were not recovered. Heart function showed greater improvement in the 100 mg gentisic acid-treated group than in the 0.5 mg bisoprolol-treated group. LVESD increased 6 weeks after TAC and significantly decreased in the 100 mg gentisic acid treatment group (Supporting Information Fig. 1A). There was no significant increase in LVEDD after TAC (Supporting Information Fig. 1B). To examine whether gentisic acid affects cardiac hypertrophy, we measured the ratio of HW/BW. As shown in Fig. 2D,E, after TAC surgery, heart size increased compared with that in the sham group and decreased after gentisic acid and bisoprolol treatment. H&E staining results further showed that the cross-sectional areas increased by TAC were reduced by gentisic acid and bisoprolol treatments (Fig. 2F,G). In particular, in the high-concentration gentisic acid group, cross-sectional area was reduced to a level similar to that in the sham group.

However, echocardiography revealed that the interventricular septal thickness at diastole (IVSd) and left ventricular posterior wall dimensions (LVPWd) also increased after TAC heart failure, when compared with the control group, but were not reduced by all drugs (Supporting Information Fig. 1C,D).

### Gentisic acid reduced the expression of cardiac hypertrophic markers in TAC-induced heart failure

**Figure 3:**
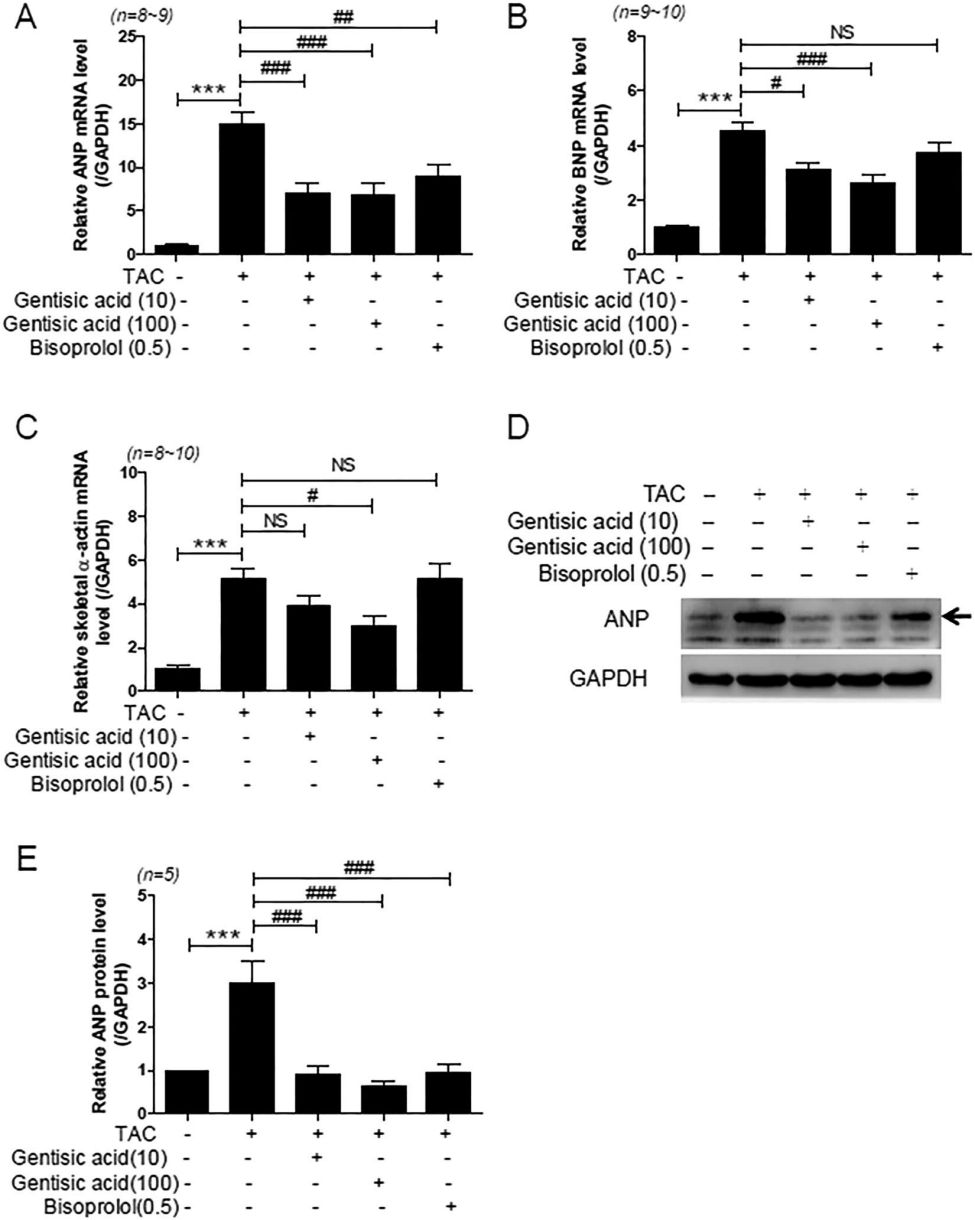
Gentisic acid reduced the expression of cardiac hypertrophic markers in TAC-induced heart failure. Three weeks after TAC, vehicle or drugs were administered for 3 weeks and heart RNA was isolated. (**A**) Atrial natriuretic peptide (ANP), (**B**) brain natriuretic peptide (BNP), and (**C**) skeletal α-actin mRNA were determined via RT-PCR. ***P < 0.001 versus the sham group; ^#^*P* < 0.05, ^##^*P* < 0.01, and ^###^*P* < 0.001 versus the TAC group; NS: not significant. (**D**) Cardiac ANP protein expression (n = 5 per group). Glyceraldehyde 3-phosphate dehydrogenase (GAPDH) was used as a loading control. (**E**) ANP protein was quantified via densitometry. Representative images were cropped from different parts of the same western blot. Full-length images are included in a Supplementary Information file. ***P < 0.001 versus the sham group; ^###^*P* < 0.001 versus the TAC group.

To determine whether cardiac hypertrophic markers were affected by gentisic acid and bisoprolol treatment, we performed RT-PCR and western blotting. The mRNA levels of ANP, brain natriuretic peptide (BNP), and skeletal α-actin significantly increased after TAC, when compared with the sham group (Fig. 3A–C). Increased ANP mRNA levels were decreased in the three treatment groups (Fig. 3A). BNP mRNA levels were dose-dependently reduced by gentisic acid treatment (Fig. 3B). However, bisoprolol treatment did not significantly reduce the expression of BNP. Administration of gentisic acid (100 mg) attenuated the expression of skeletal α-actin mRNA after TAC. In addition, ANP protein levels were significantly reduced in the three treatment groups (Fig. 3D,E).

### Gentisic acid decreased expression of RAAS components in TAC-induced heart failure

**Figure 4:**
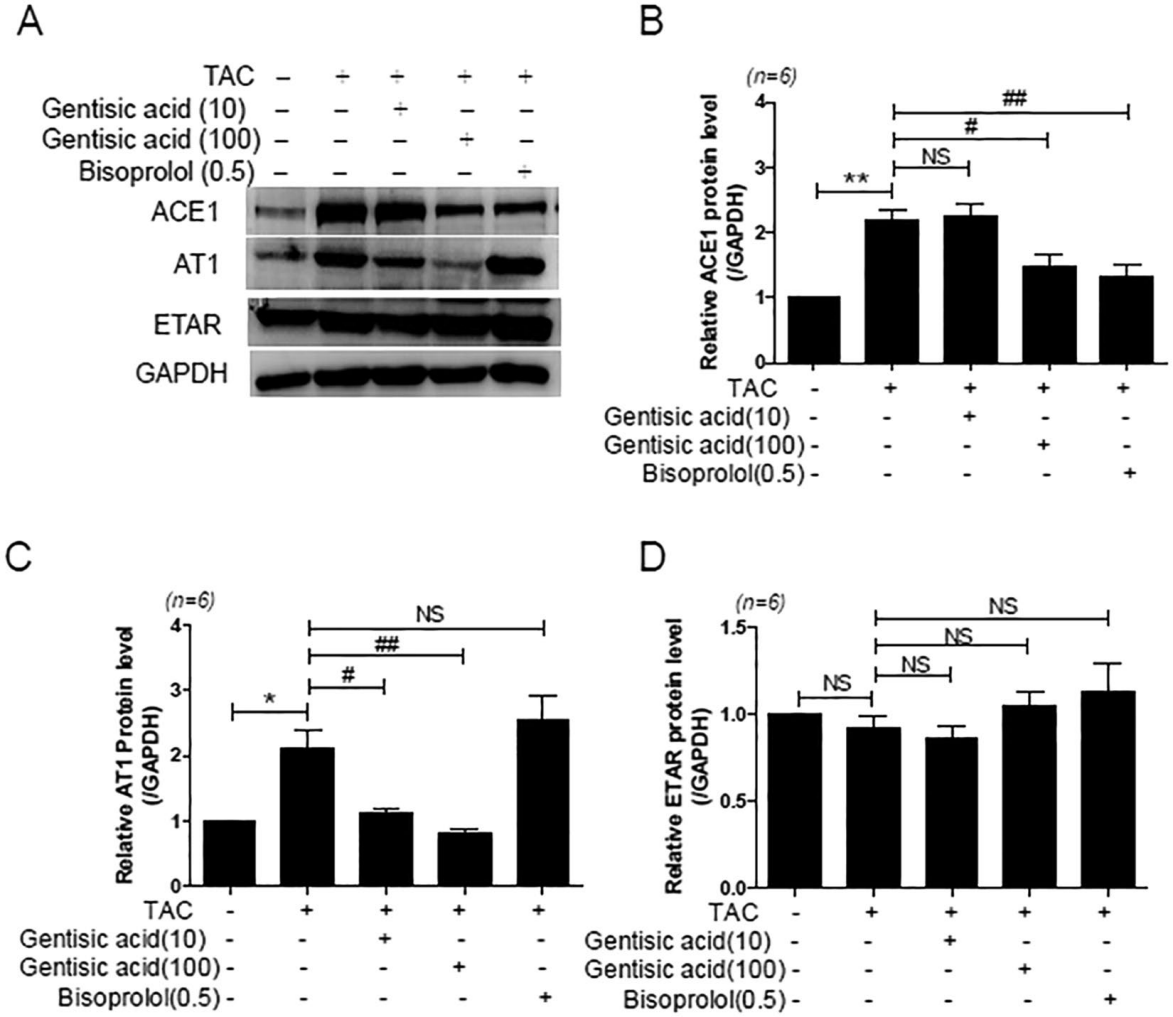
Gentisic acid reduced the expression of angiotensin-converting enzyme 1 (ACE1) and angiotensin II receptor type 1 (AT1) in TAC-induced heart failure. Three weeks after TAC, mice were administered the vehicle, gentisic acid (10 mg∙kg^−1^∙day^−1^ or 100 mg∙kg^−1^∙day^−1^), or bisoprolol (0.5 mg∙kg^−1^∙day^−1^) daily for 3 weeks. (**A**) Representative western blots of ACE1, AT1, and endothelin-1 receptor type A (ETAR) expression in hearts from mice in the sham, TAC, TAC + gentisic acid (10 mg∙kg^−1^∙day^−1^ or 100 mg∙kg^−1^∙day^−1^), or TAC + bisoprolol (0.5 mg∙kg^−1^∙day^−1^) groups. (**B**‒**D**) The protein levels of ACE, AT1, and ETAR were quantified. Representative images were cropped from different parts of the same western blot. Full-length images are included in a Supplementary Information file. *P < 0.05 and **P < 0.01 versus the sham group; ^#^*P* < 0.05 and ^##^*P* < 0.01 versus the TAC group; NS: not significant.

To clarify the regulatory mechanism of the inhibitory action of gentisic acid on TAC-induced heart failure, we examined heart RAAS components. The expression of ACE1 and AT1 was higher in the TAC group than in the sham group (Fig. 4A). ACE1 levels decreased significantly after treatment with 100 mg gentisic acid and bisoprolol (Fig. 4B). AT1 expression was not reduced by bisoprolol treatment but decreased after gentisic acid treatment in a dose-dependent manner (Fig. 4C). The increase of endothelin-1 in plasma and hearts is associated with heart failure^24^. However, ETAR expression was unchanged in response to TAC or drug treatment (Fig. 4A,D).

### Gentisic acid inhibited cardiac fibrosis in TAC-induced heart failure

**Figure 5:**
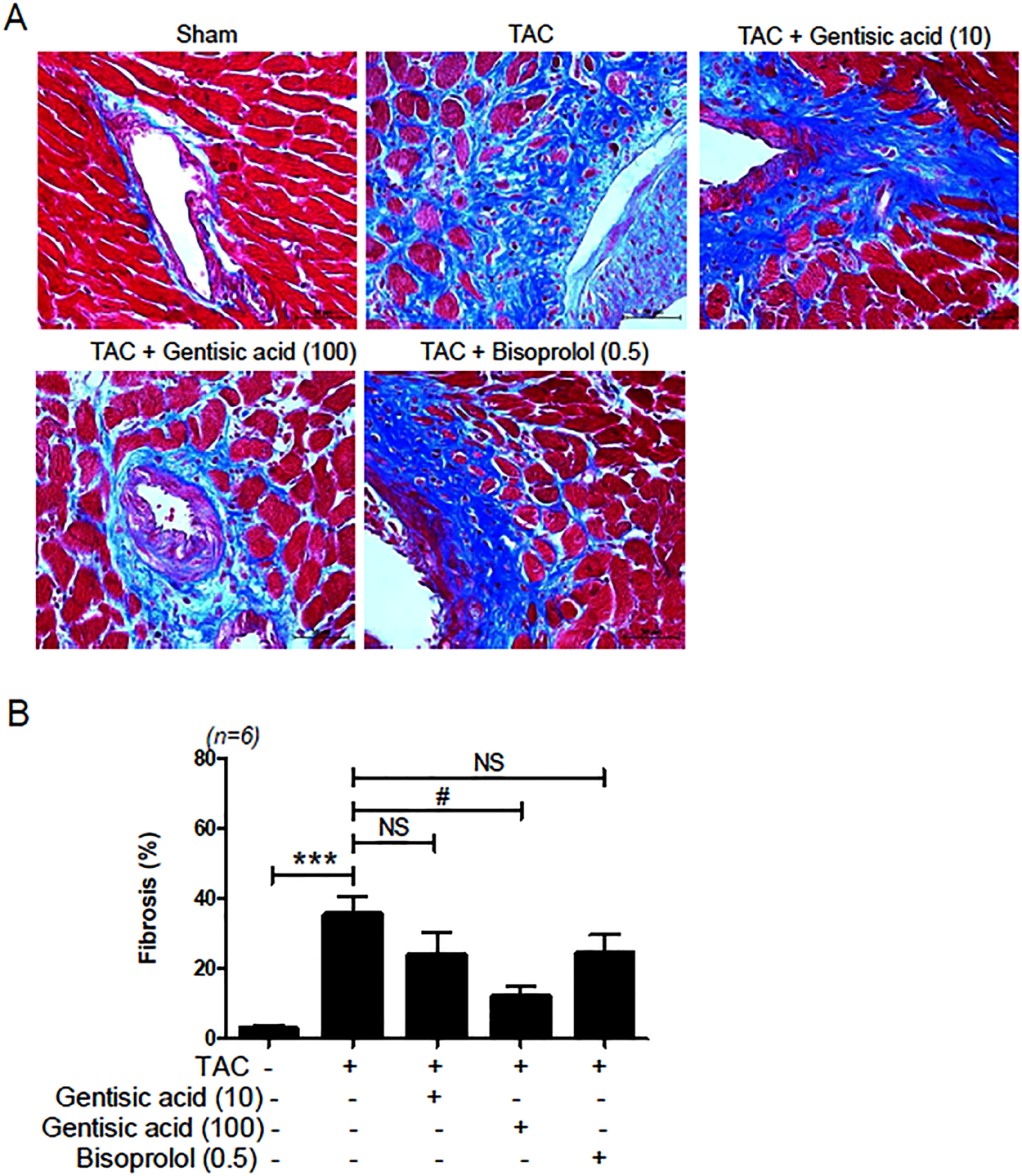
Gentisic acid attenuated cardiac fibrosis in TAC-induced heart failure. Three weeks after TAC, mice were administered the vehicle, gentisic acid, or bisoprolol daily for 3 weeks. (**A**) Representative images of hearts stained with Masson’s trichrome from the sham, TAC, TAC + gentisic acid (10 mg∙kg^−1^∙day^−1^), TAC + gentisic acid (100 mg∙kg^−1^∙day^−1^), or bisoprolol (0.5 mg∙kg^−1^∙day^−1^) groups. Collagen deposits were stained blue. Scale bar = 50 μm. (**B**) Quantitative data of Masson’s trichrome staining for interstitial and perivascular collagen deposits in hearts (n = 6 per group). ***P < 0.001 versus the sham group; ^#^*P* < 0.05 versus the TAC group; NS: not significant.

Fibrosis is characteristic of heart failure. Here, we examined whether gentisic acid prevents cardiac fibrosis. Masson’s trichrome staining revealed prominent collagen deposits in the TAC group, when compared with the sham group, especially in the perivascular region (Fig. 5A). Total fibrosis, including interstitial and perivascular fibrosis, increased significantly after TAC (Fig. 5B). Fibrosis was inhibited by treatment with the highest concentration of gentisic acid, but bisoprolol treatment did not cause any significant decrease.

**Figure 6:**
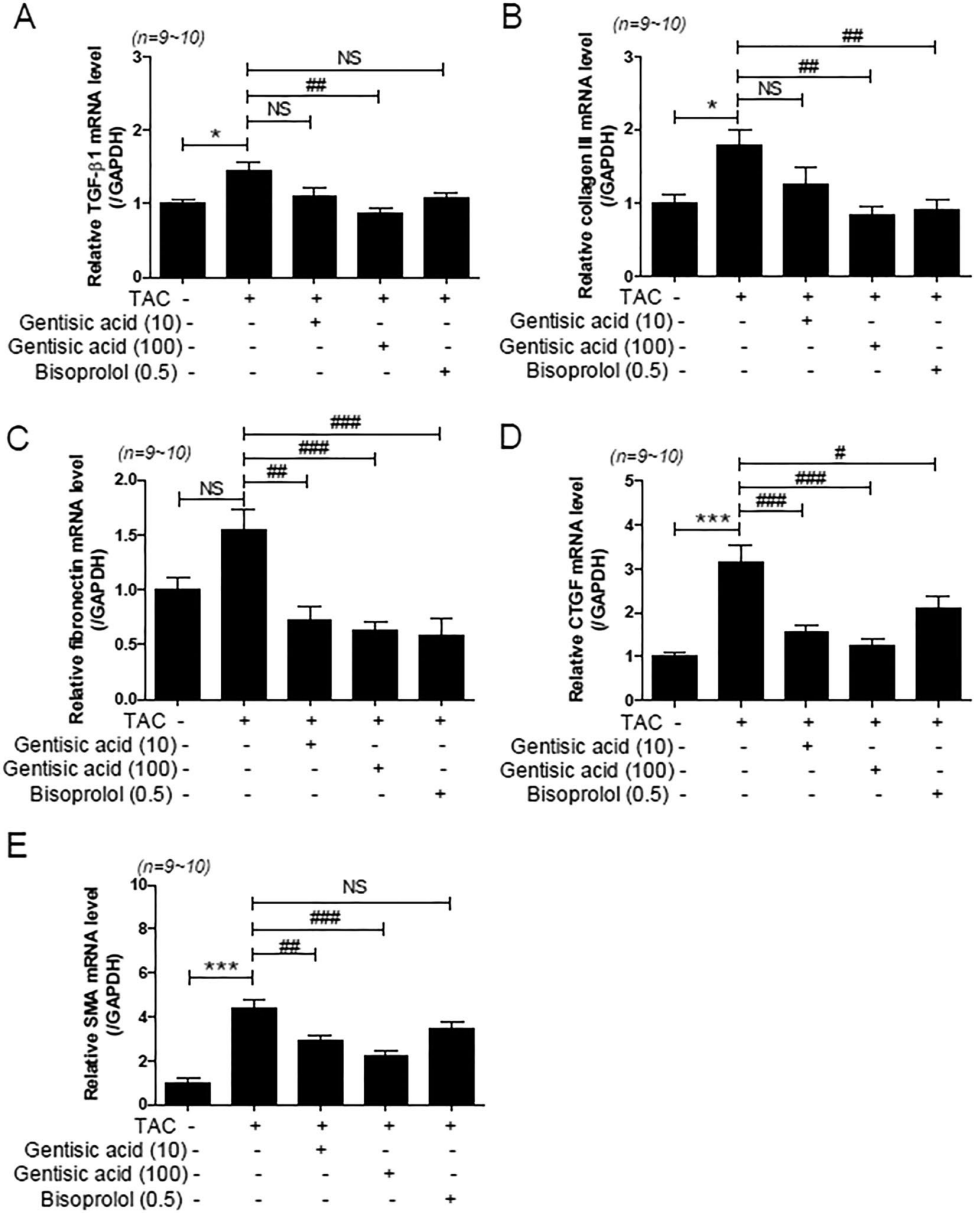
Gentisic acid ameliorated mRNA expression of cardiac fibrosis marker-related genes in TAC-induced heart failure. Three weeks after TAC, mice were administered the vehicle, gentisic acid (10 mg∙kg^−1^∙day^−1^ or 100 mg∙kg^−1^∙day^−1^), or bisoprolol (0.5 mg∙kg^−1^∙day^-1^) daily for 3 weeks. **(A**‒**E**) mRNA levels of transforming growth factor beta-1 (TGF-β1), collagen III, fibronectin, connective tissue growth factor (CTGF), and smooth muscle alpha actin (SMA) were analyzed via RT-PCR (n = 9–10 per group). *P < 0.05 and ***P < 0.001 versus the sham group; ^#^*P* < 0.05, ^##^*P* < 0.01, and ^###^*P* < 0.001 versus the TAC group; NS: not significant.

To investigate whether gentisic acid affects fibrosis marker gene expression (TGF-β1, collagen III, fibronectin, CTGF, and SMA), we conducted RT-PCR and western blotting. The expression of four fibrosis markers, excluding fibronectin, was significantly increased in the TAC group compared with that in the sham group. Higher concentration of gentisic acid (100 mg∙kg^−1^∙day^−1^) reduced expression of all increased markers (Fig. 6A–E). However, treatment with 10 mg∙kg^−1^∙day^−1^ gentisic acid and bisoprolol (0.5 mg∙kg^−1^∙day^−1^) was less effective than that observed with higher concentrations of gentisic acid.

**Figure 7:**
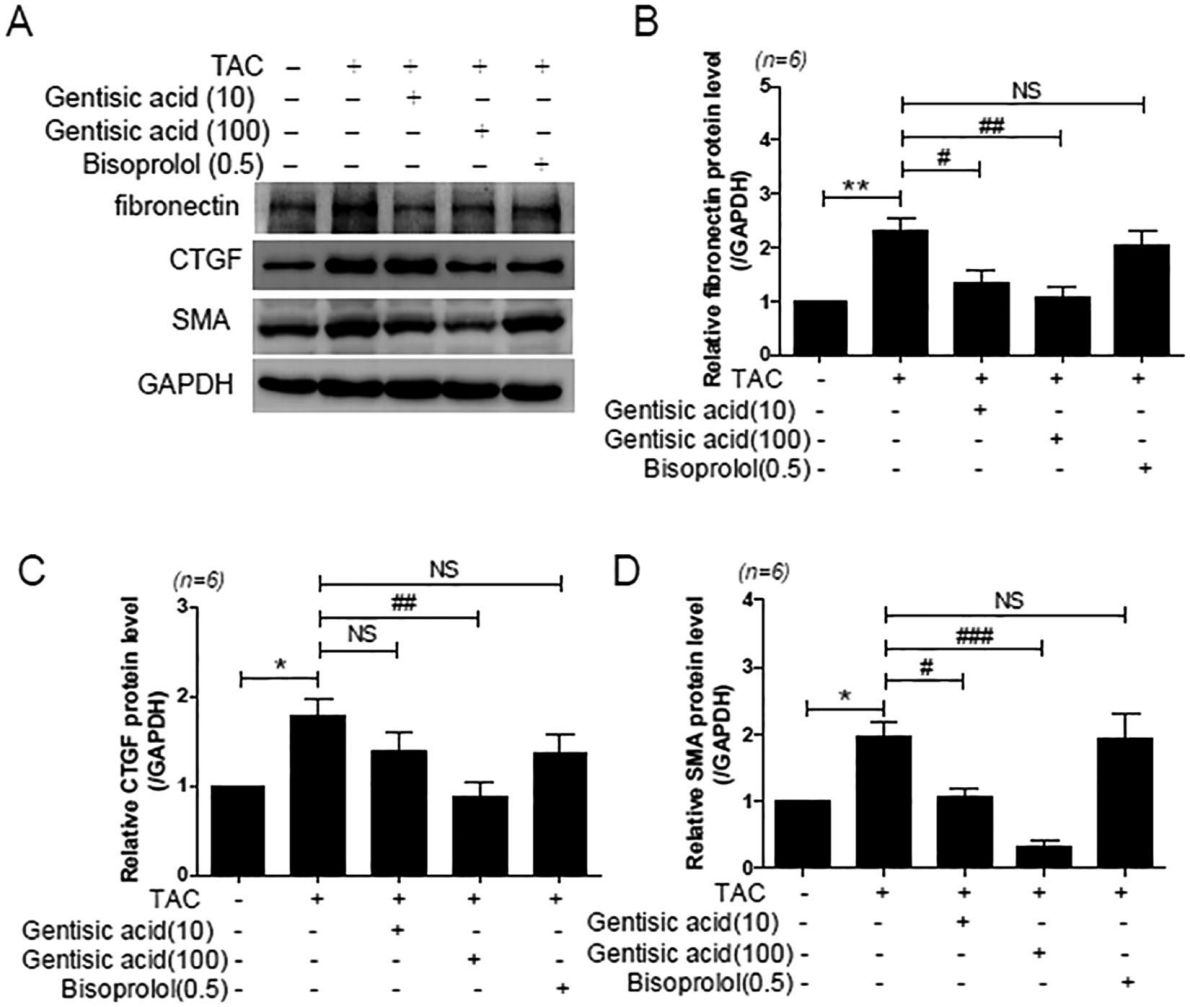
Gentisic acid reduced cardiac fibrosis marker protein expression in TAC-induced heart failure. Three weeks after TAC, mice were administered the vehicle, gentisic acid (10 mg∙kg^−1^∙day^−1^ or 100 mg∙kg^−1^∙day^−1^), or bisoprolol (0.5 mg∙kg^−1^∙day^−1^) daily for 3 weeks. (**A**) Representative western blot images for fibronectin, CTGF, and SMA protein expression. (**B**–**D**) Protein levels of fibronectin, CTGF, and SMA were quantified via densitometry. Representative images were cropped from different parts of the same or different western blots. Full-length images are included in a Supplementary Information file. The data are expressed as means ± SEM (n = 6 per group).

We also investigated the expression of fibrosis-related genes via western blotting. Similarly, cardiac expression of fibronectin, CTGF, and SMA was significantly higher in the TAC group than in the sham group (Fig. 7A). Treatment with high-concentration gentisic acid reduced the expression of fibronectin, CTGF, and SMA, whereas bisoprolol treatment did not affect these (Fig. 7B–D).

We further determined the expression of other fibrosis-associated genes. Lysyl oxidase (LOX), LOX-like 1 (LOXL1), and LOXL2 were upregulated after TAC^25^. Unlike previous reports, our experiments showed no change in gene expression (Supporting Information Fig. 2A–C). WNT1 inducible signaling pathway protein 2 (CCN5), a transcriptional repressor of TGF-β, was reported to be down-regulated in failing hearts^26^. However, CCN5 mRNA levels were not reduced after TAC (Supporting Information Fig. 2D). Mothers against decapentaplegic homolog 7 (SMAD7) is known to attenuate TGF-β signaling^27^. The expression of SMAD7 did not differ between groups (Supporting Information Fig. 2E).

### Gentisic acid reduced pulmonary vascular remodeling and TAC-induced heart failure-associated increased LW

**Figure 8:**
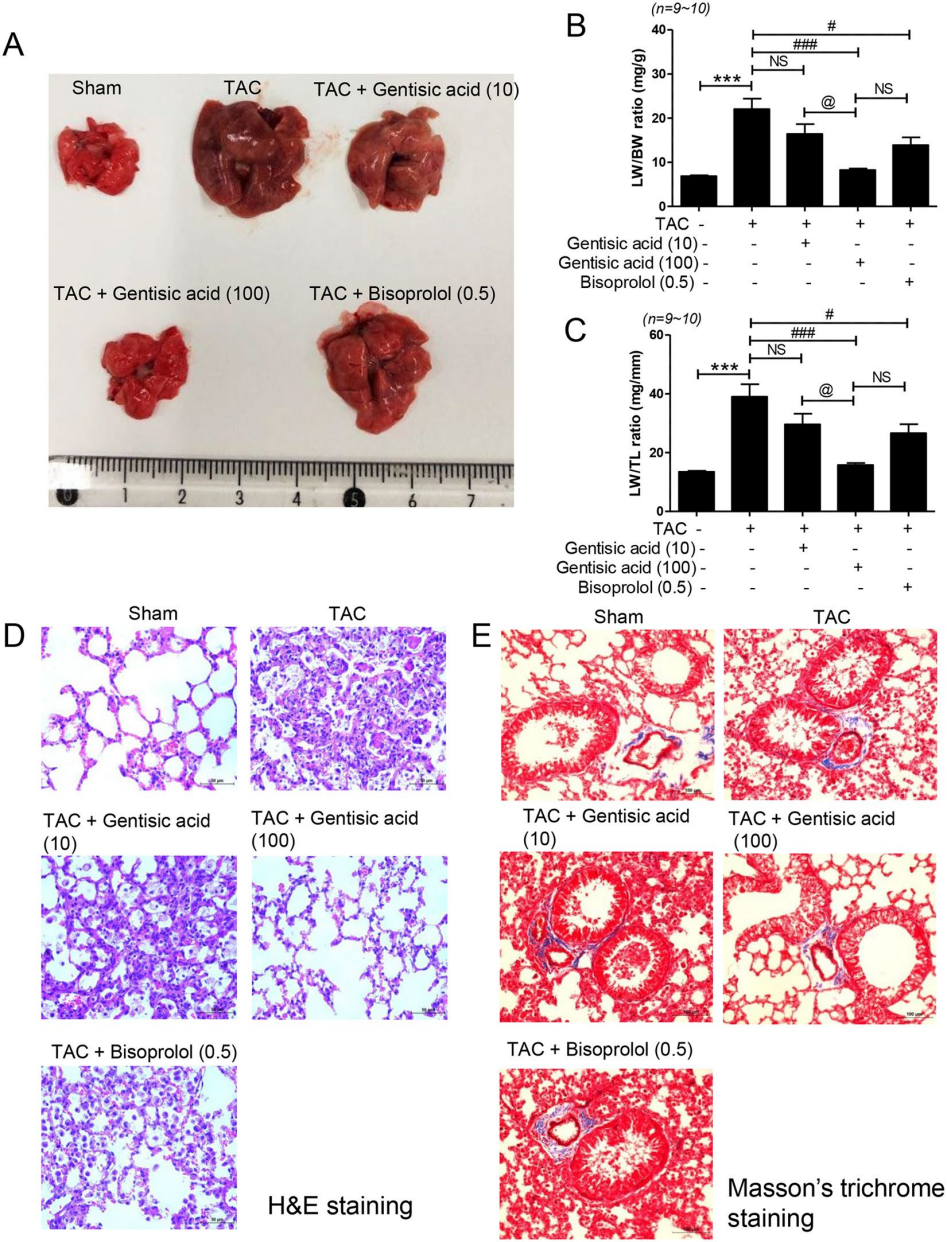
Gentisic acid reduced the weight of the increased lungs and type II pneumocyte hyperplasia in TAC-induced heart failure. Three weeks after TAC, mice were administered the vehicle, gentisic acid (10 mg∙kg^−1^∙day^−1^ or 100 mg∙kg^−1^∙day^−1^), or bisoprolol (0.5 mg∙kg^−1^∙day^−1^) daily for 3 weeks. (**A**) Representative lung photographs. One ruler column represents 1 mm. (**B**) Ratio of lung weight to body weight and (**C**) the ratio of lung weight to tibia length. ***P < 0.001 versus the sham group; ^#^*P* < 0.05 and ^###^*P* < 0.001 versus the TAC group; ^@^*P* < 0.05 versus the gentisic acid-treated group (100 mg∙kg^−1^∙day^−1^); NS: not significant. (**D**) Representative H&E-stained images from the sham, TAC, TAC + gentisic acid (10 mg∙kg^−1^∙day^−1^), TAC + gentisic acid (100 mg∙kg^−1^∙day^−1^), and TAC + bisoprolol (0.5 mg∙kg^−1^∙day^−1^) groups. Scale bar = 50 μm. (**E**) Representative Masson’s trichrome staining of lung tissue sections. Scale bar = 100 μm.

TAC-induced heart failure is associated with increased LW^28,29^. Therefore, we investigated whether gentisic acid treatment affected LW in the TAC group. LW/BW and LW/TL ratios were significantly increased in the TAC group than in the sham group (Fig. 8A–C). The TAC-induced heart failure group showed more than three-fold increases in LW, although this increase was recovered by treatment with gentisic acid (100 mg∙kg^−1^∙day^−1^). However, treatment with 10 mg gentisic acid did not significant reduce LW, when compared with the sham group. The gentisic acid-associated reduction in LW/BW and LW/TL was dose-dependent.

Bisoprolol treatment reduced LW in the TAC group (Fig. 8B,C). To further investigate the morphological changes in lung tissues, H&E staining was carried out. The lung tissues of the sham group revealed normal open alveolar spaces (Fig. 8D). Type II pneumocyte hyperplasia was observed in the lungs of mice in the TAC group but not in those of the sham group (Fig. 8D). In the group treated with 100 mg∙kg^−1^∙day^−1^ gentisic acid, air spaces were well maintained and no hyperplasia was observed. However, there was limited type II pneumocyte hyperplasia observed in the low-concentration gentisic acid (10 mg∙kg^−1^∙day^−1^) and bisoprolol-treated groups.

To investigate whether gentisic acid affects lung fibrosis in TAC-induced heart failure, Masson’s trichrome staining and RT-PCR were performed. Interestingly, the TAC group did not show collagen deposition (Fig. 8E). We investigated changes in fibrosis markers to further confirm that TAC lungs did not show fibrosis. Unexpectedly, mRNA expression of collagen I, collagen III, fibronectin, and CTGF were significantly decreased when compared with the sham group. There was no significant difference in expression between the drug treatment groups (Supplementary Fig. 3A–D).

## Discussion

Here, we showed that gentisic acid prevents the transition from cardiac hypertrophy to heart failure in a pressure-overload mouse model. These data demonstrate that gentisic acid is superior to the clinically used BB bisoprolol. Treatment with 100 mg∙kg^−1^∙day^−1^ gentisic acid reduced cardiac hypertrophy and fibrosis and prevented the deterioration of heart function. Interestingly, gentisic acid administration (100 mg∙kg^−1^∙day^−1^) maintained EF and FS values that were similar to those of the control group. The conclusion that gentisic acid prevents cardiac dysfunction is an important finding that suggests its potential as an agent in the prevention or treatment of heart failure. Low-concentration gentisic acid and bisoprolol treatment did not improve heart function or prevent cardiac fibrosis as determined by Masson’s trichrome staining. However, bisoprolol was able to partially reduce heart size, which indicates that a concentration of 0.5 mg∙kg^−1^∙day^−1^ is not enough to restore cardiac dysfunction. Bisoprolol is administered at a maximum dose of 10 mg per day in adults, which corresponds to 0.14 mg∙kg^−1^ in animals. In this study, we used 0.5 mg∙kg^−1^, which is higher than that used in clinical practice. The effect of gentisic acid on the prevention of heart dysfunction and fibrosis is also dose-dependent (Figs 2B and 5B). Our recent report showed that gallic acid with structure similar to gentisic acid improves heart failure^23^. However, there are some differences between the previous and current studies; the effect of the drugs (gallic acid vs gentisic acid) was different from the therapeutic and preventive perspectives. Thus, the timing of drug administration in this study was changed from 8 weeks to 3 weeks after TAC surgery.

Safety should be the first consideration when using high concentrations of gentisic acid in clinical trials. The acute oral toxicity results were similar in the gentisic acid treatment group and the control group. No weight changes or pathological findings were observed in the liver, lungs, kidneys, and heart. Blood tests may reveal differences in ALP, and basophil and platelet counts, but they do not indicate the impact on tissue damage. Upregulation of ANP, BNP, and skeletal α-actin has been reported in cardiac hypertrophy and heart failure^30,31^. Indeed, the expression of these genes was increased in the TAC group and decreased after gentisic acid treatment. Like ANP, plasma BNP and N-terminal (NT)-proBNP concentrations in acute heart failure were higher than those in healthy people^32^. Plasma BNP levels were inversely correlated with the left ventricular EF and end diastolic dimensions. Furthermore, BNP levels were reduced by treatment with drugs such as BB, ACE inhibitors, vasodilators, ARB, and others^33^. Sustained activation of neurohormonal systems such as the SNS and the RAAS is regarded as one of the mechanisms supporting the development of heart failure^34^. ACE inhibitors and BB are known to be the most important drugs to inhibit or prevent heart failure^11,35,36^. Furthermore, cardiac ACE1 is upregulated in congestive heart failure^37^, implying that ACE is associated with heart failure. In agreement with a previous report on TAC^38^, we confirmed that the expression of ACE1 and AT1 in TAC-induced heart failure was increased compared with that in the control group. Gentisic acid treatment significantly reduced the expression of these two proteins, suggesting that gentisic acid contributes to preventing heart failure through attenuation of the RAAS. Bisoprolol administration reduced the expression of ACE1 in TAC-induced heart failure, but did not affect that of AT1. Bisoprolol has beneficial effects on mortality and morbidity in patients with stable chronic heart failure^39^. This study is the first to report that gentisic acid is more effective than bisoprolol in preventing heart failure. The endothelin system is activated in congestive heart failure^40^. However, the expression of ETAR was not induced in TAC.

Fibrosis, along with cardiac hypertrophy, is a common feature of heart failure^41,42^. Increased collagen deposition in and around the vessels and interstitial myocardium can impair cardiac function. Inhibiting fibrosis is therefore an important target for the treatment of heart failure. Here, characteristic fibrosis occurred 6 weeks after TAC in the heart but not in the lungs. Treatment with 100 mg gentisic acid was the most effective in preventing collagen deposition as determined by Masson’s trichrome staining. Fibrosis inhibition by gentisic acid was dose-dependent. The mRNA or protein expression of fibrosis-related markers was also significantly reduced by gentisic acid treatment in a dose-dependent manner.

In our previous study^29^, lung fibrosis was evident 10 weeks after TAC surgery. In this TAC-induced heart failure model, the increase in LW preceded fibrosis. Masson’s trichrome staining revealed no fibrosis in the lungs 6 weeks TAC. Surprisingly, the mRNA levels of fibrosis markers were significantly lower than those in the control group (Fig. 8E and Supporting Information Fig. 3).

In the present study, the most interesting finding is that TAC-associated increase in LW was inhibited by gentisic acid treatment. In addition, the gentisic acid treatment group did not show any abnormal lung structures after TAC. Typically, type II pneumocyte hyperplasia appears in acute and chronic lung injury^43^. We demonstrated that gentisic acid inhibited hyperplasia of type II pneumocytes. These results suggest that gentisic acid is a strong candidate phytochemical that can prevent or treat lung disease. The present study had some limitations. First, although cardiac hypertrophic markers have been evaluated extensively, we did not measure plasma BNP and NT-proBNP levels. Second, although enlarged lung size is associated with lung edema in TAC, we did not determine the wet and dry weight of the lungs. Third, although activation of the neurohormonal system is closely related to heart failure, we did not assess the SNS. In the future, thorough investigations should be conducted on the underlying mechanisms. Furthermore, it is necessary to use relatively low-dose gentisic acid in clinical studies to prevent toxicity.

This is the first report demonstrating that gentisic acid can prevent the transition from cardiac hypertrophy to heart failure with abnormal lung structures. Gentisic acid prevents cardiac dysfunction and fibrosis and inhibits the RAAS. We suggest that gentisic acid can be useful for the prevention or treatment of heart failure with relatively low toxicity.

## Supplementary information

Supplementary Figures and western blot full blots
