# Supplementary material for: Gentisic acid prevents the transition from pressure overload-induced cardiac hypertrophy to heart failure
Source: Sci Rep. 2019 Feb 28;9:3018. doi: 10.1038/s41598-019-39423-8 (PMC6395621; doi:10.1038/s41598-019-39423-8)
Supplement: Supplementary file 1 — Supplementary Figures and western blot full blots [file 41598_2019_39423_MOESM1_ESM.pdf]

Gentisic acid prevents the transition from pressure overload-induced cardiac hypertrophy to heart failure

Simei Sun[1,2,3,4], Hae Jin Kee[2,3*], Yuhee Ryu[2,3], Sin Young Choi[2,3,4], Gwi Ran Kim[2,3], Hyung-Seok Kim[5], Seung-Jung Kee[6], and Myung Ho Jeong[2,3*]

[1]Zhoushan Hospital, Zhejiang University School of Medicine, No 739, Dingshen Road Lincheng New District Zhoushan Zhejiang 316021, China

[2]Heart Research Center of Chonnam National University Hospital, Gwangju 61469, Republic of Korea

[3]Hypertension Heart Failure Research Center, Chonnam National University Hospital, Gwangju 61469, Republic of Korea

[4]Molecular Medicine, BK21 plus, Chonnam National University Graduate School, Gwangju 61469, Republic of Korea

[5]Department of Forensic Medicine, Chonnam National University Medical School, Gwangju 61469, Republic of Korea

[6]Department of Laboratory Medicine, Chonnam National University, Medical School and Hospital, Gwangju 61469, Republic of Korea

Supplementary figure legends

Supplementary Figure 1. Echocardiographic parameters of mice subjected to transverse aortic constriction (TAC)

Echocardiographic parameters were assessed 6 weeks after TAC surgery. (**A**) Left ventricular end-systolic diameter (LVESD), (**B**) left ventricular end-diastolic diameter (LVEDD), (**C**) interventricular septum thickness (IVSd); (**D**) left ventricular posterior wall thickness (LVPWd).

Supplementary Figure 2. Expression of fibrosis-associated markers in heart tissue after TAC

Levels of lysyl oxidase (LOX, **A**), lysyl oxidase-like 1 (LOXL1, **B**), LOXL2 (**C**), WNT1 inducible signaling pathway protein 2 (CCN5, **D**), and mothers against decapentaplegic homolog 7 (SMAD7, **E**) mRNA were evaluated via reverse transcription polymerase chain reaction (RT-PCR). Data are means ± standard error of the mean (SEM). NS, not significant.

Supplementary Figure 3. Fibrosis marker gene expression in lung tissue after TAC

Transcript levels for collagen type I (**A**), collagen type III (**B**), fibronectin (**C**), and connective tissue growth factor (CTGF, **D**) were evaluated via RT-PCR. Data are means ± SEM. ***$P < 0.001$ versus the sham group; NS, not significant.

Supporting Information Figure 1

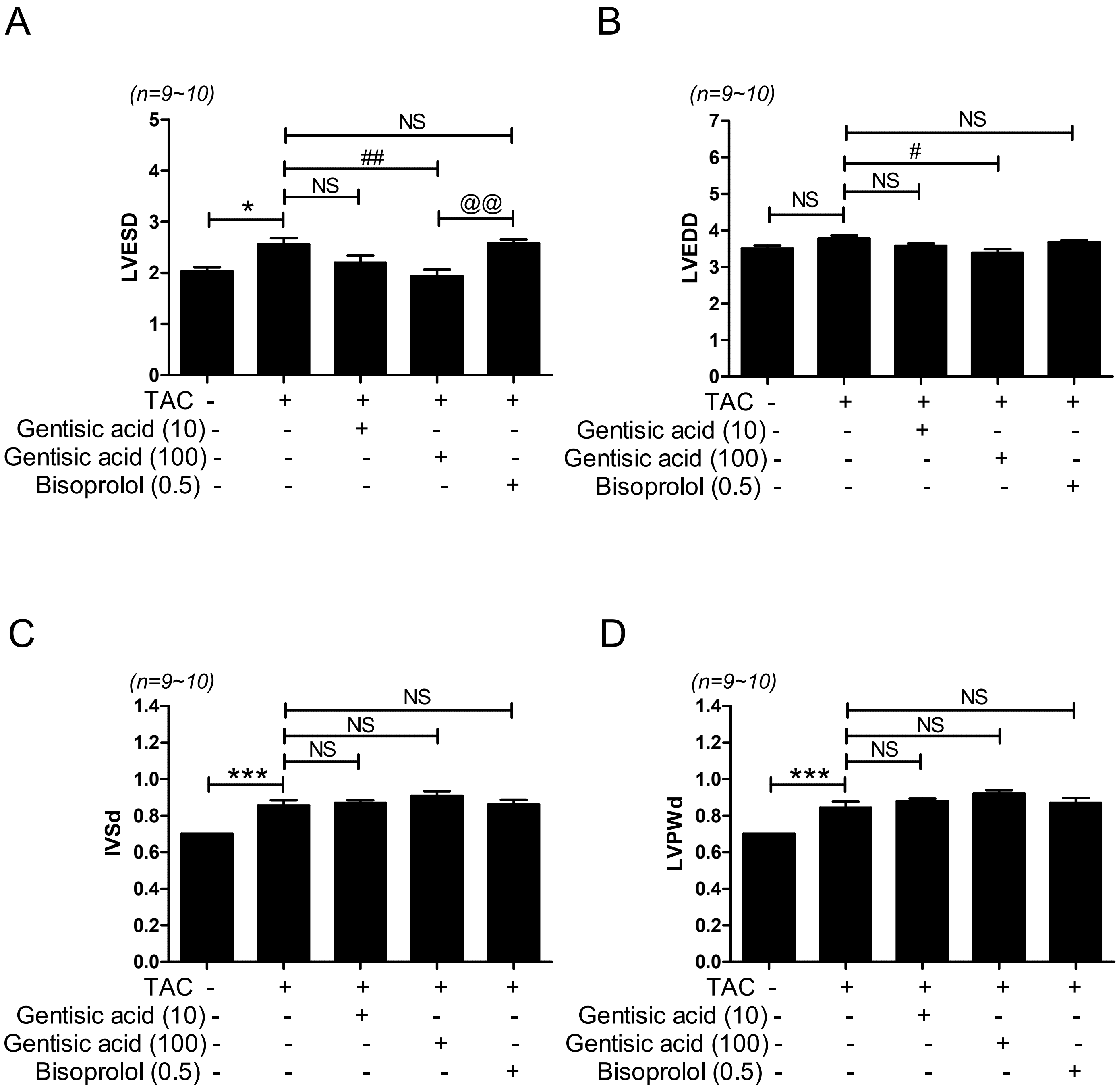

Supporting Information Figure 2

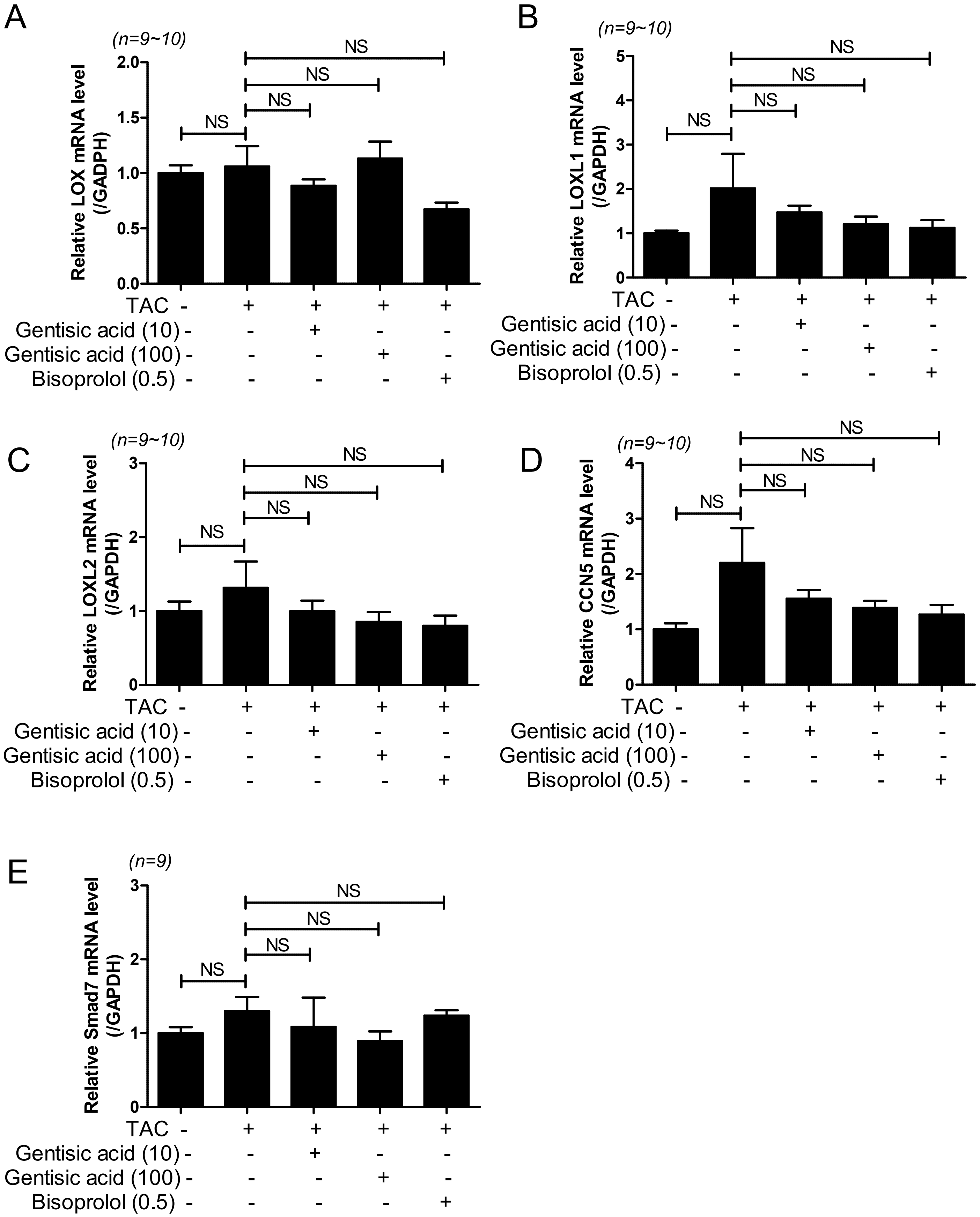

Supporting Information Figure 3

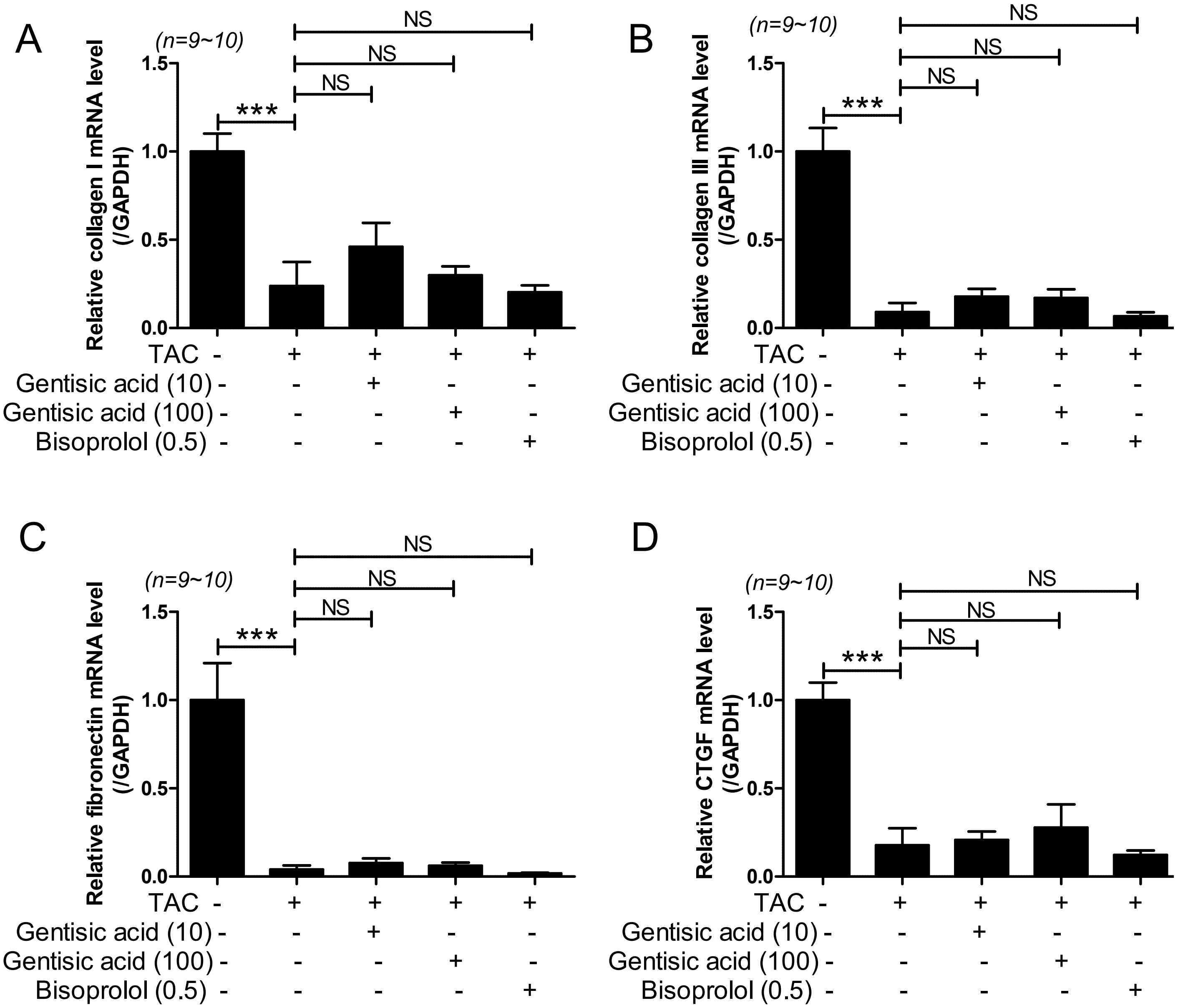

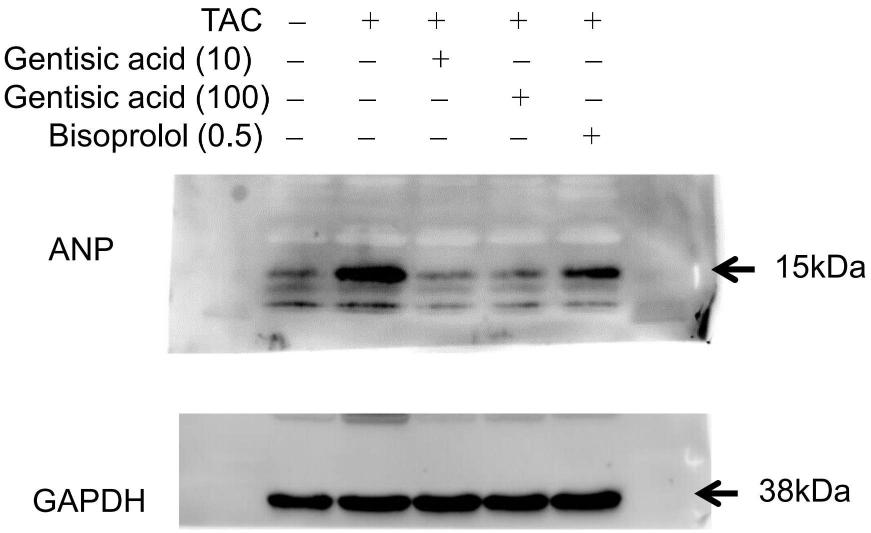
TAC – + + + +
Gentisic acid (10) – – + – –
Gentisic acid (100) – – – + –
Bisoprolol (0.5) – – – – +
ANP
15kDa
GAPDH
38kDa

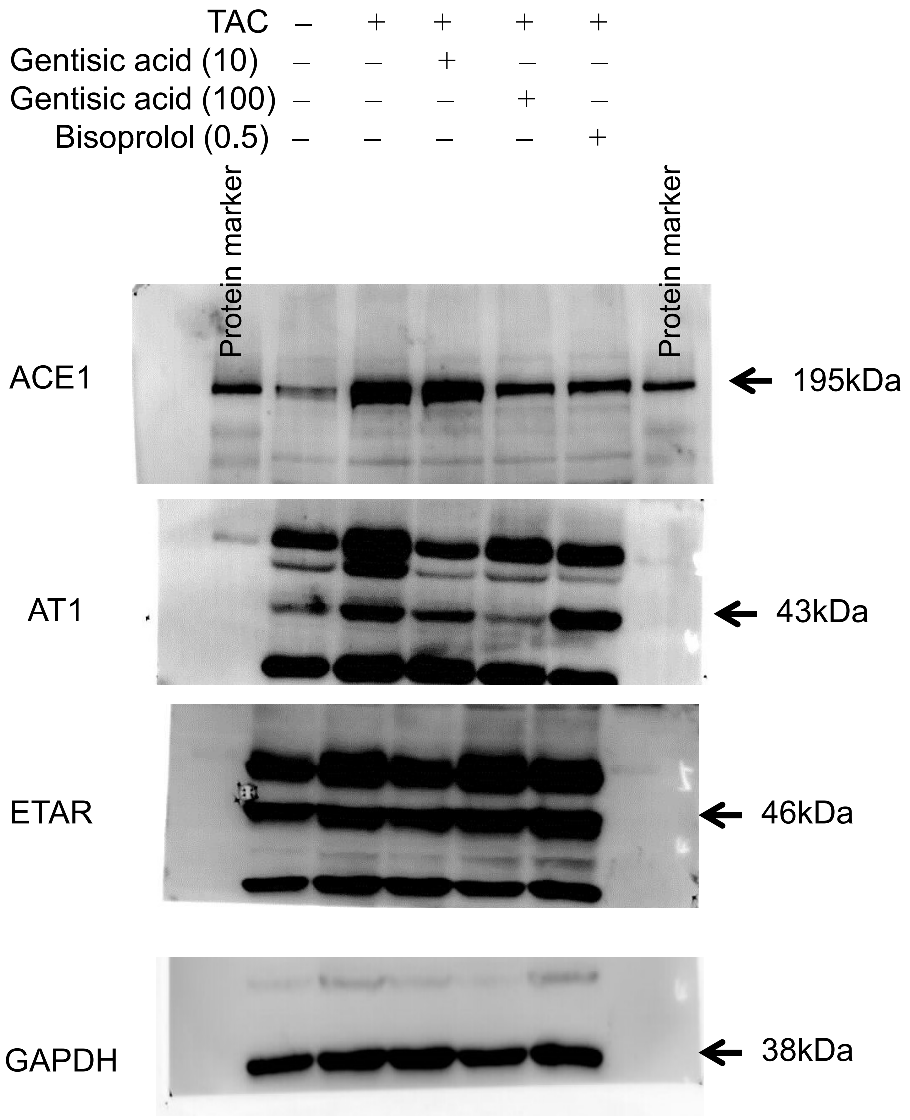
TAC – + + + +
Gentisic acid (10) – – + – –
Gentisic acid (100) – – – + –
Bisoprolol (0.5) – – – – +
Protein marker
Protein marker
ACE1
195kDa
AT1
43kDa
ETAR
46kDa
GAPDH
38kDa

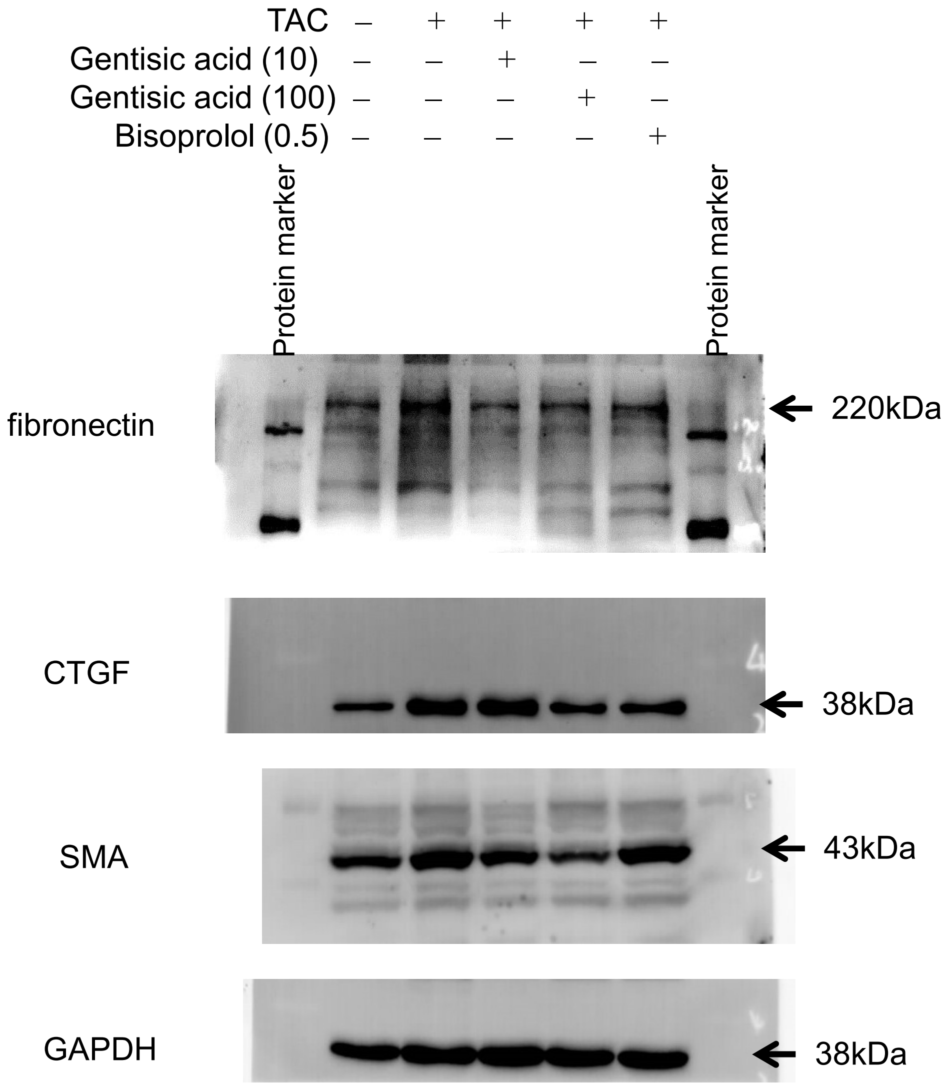
TAC – + + + +
Gentisic acid (10) – – + – –
Gentisic acid (100) – – – + –
Bisoprolol (0.5) – – – – +
Protein marker
Protein marker
fibronectin
220kDa
CTGF
38kDa
SMA
43kDa
GAPDH
38kDa
